# Wheat and Oat Brans as Sources of Polyphenol Compounds for Development of Antioxidant Nutraceutical Ingredients

**DOI:** 10.3390/foods10010115

**Published:** 2021-01-07

**Authors:** Ana Belén Martín-Diana, María Jesús García-Casas, Cristina Martínez-Villaluenga, Juana Frías, Elena Peñas, Daniel Rico

**Affiliations:** 1 Subdirection of Research and Technology, Agro-Technological Institute of Castilla y León, Consejería de Agricultura y Ganadería, Finca de Zamadueñas, Ctra. Burgos Km. 119, 47071 Valladolid, Spain; garcasmj@itacyl.es (M.J.G.-C.); ricbarda@itacyl.es (D.R.); 2 Department of Food Characterization, Quality and Safety, Institute of Food Science, Technology and Nutrition (ICTAN-CSIC), Juan de la Cierva 3, 28006 Madrid, Spain; c.m.villaluenga@csic.es (C.M.-V.); frias@ictan.csic.es (J.F.); elenape@ictan.csic.es (E.P.)

**Keywords:** wheat, oat, bran, antioxidant, ferulic acid, lignin, glycemic index

## Abstract

Bran, a byproduct still mainly used for animal feed, is receiving increased attention as potential ingredient for a healthier diet. The aim of this study was to characterize and evaluate the nutritional and antioxidant properties of wheat and oat bran in order to promote their use as nutraceutical ingredients in flour and/or other products. The effects of grain (wheat vs. oat) and milling fraction (whole grain vs. bran) on the phenolic profile (free vs. bound phenolics), antioxidant and nutrient profiles, and glycemic index were evaluated. Differences in antioxidant capacity through different methodologies between grain and bran were observed, supporting a higher in vitro antioxidant capacity of the whole grain than that of the refined flours, which lack the bran fraction. The highest RACI (Relative Antioxidant Capacity Index) corresponded to wheat bran bound fraction, which showed the highest concentration of ferulic acid and correlation with antioxidant parameters tested. The in vitro glycemic index of the bran fractions was reduced, as compared with grain, with lower values found for wheat. The results support the important benefits of the polyphenols linked to fiber and the importance to develop methods to increase bioavailability of these compounds, which would promote WB use as nutraceutical ingredient.

## 1. Introduction

Cereals are staple food for most of the world’s population. Extensive literature supports the consumption of whole grain-products over refined flours, as the first provide improved gut health, immunity, and weight control due to their nutritional and bioactive properties [1,2,3,4,5]. Whole-grain consumption seems to have benefits not only for adults, but also for children. Damsgaard et al. [6] showed that the consumption of whole grains in early ages can contribute to control insulin levels, adequately reducing diabetes, obesity, and incidence of related diseases during infancy or adult ages.

In addition to fiber-related benefits of whole grains, which have been extensively reported [7], research has shown further benefits related to the presence of biologically active compounds and to synergistic effects between dietary fiber and different micronutrients [7,8]. The European Food Safety Authority recommends a dietary fiber intake of 25 g/day, ranging from 10 to 20 g/day in young children and from 16 to 29 g/day in adults [9]. For children, this varies and the general rule for health care professionals is to calculate it as age of child plus 5 (g/day).

One important limitation to the use of whole grain flours is associated with their impact on the techno-functional properties of doughs and sensorial attributes of the final products, affecting aspects such as structure, texture, color, and general acceptability, which has favored the consumption of refined flours over decades. However, this consumption trend has been steadily changing over the last years, which is mostly related to the increasing evidence associating wholegrain consumption and health benefits [10,11,12,13,14,15]. Low availability of wholegrain meeting consumer expectations has been pointed out as an obstacle to increase wholegrain consumption [16]. Understanding the differences in expected attributes of the products formulated with refined or wholegrain flours is important in order to facilitate the technical development of more attractive wholegrain products [17].

Wheat grows under various climatic conditions and has been a staple food of the major civilizations in Europe, Asia, and North Africa for over 8000 years, used in a wide variety of products and post-production operations, and playing an important role in creating a stable supply of several products [18]. Oat is a cool season crop that has been used as a foodstuff for both humans and livestock for millennia. It is a staple crop in Russia, Canada, US, Germany, Ireland, and Scotland, and ranks sixth in the cereal production after maize, wheat, rice, barley, and sorghum [19].

Bran obtained from whole grains is an excellent source of fiber that can be used to balance the deficiency in flour formulations, improving functionality. Bran is defined as the edible broken coat, or protective outer layer of cereal grains, separated from the kernel. In flour processing, bran is removed from the ground kernels by sifting or bolting in a rotating, meshed, cylindrical frame [20]. Most of the bran is destined to animal feed [21] and has traditionally received less attention with respect to its nutritional, phytochemical, and functional properties, so its use for human consumption has been based on specific applications, being appreciated mainly for its high content of insoluble fiber [22].

Bran from whole cereal grains such as wheat and oat is predominantly composed of non-starch polysaccharides (NSPs, ∼38%), starch (∼19%), protein (∼18%), and lignin (∼6%), with the NSPs being primarily arabinoxylans (∼70%), cellulose (∼19%), and β-(1,3)/β-(1,4)-glucan (∼6%). Wheat bran (WB) typically contains approximately 45% of dietary fiber, of which about 95% is insoluble fiber [23,24]. WB bolsters digestive health by providing an important source of insoluble fiber, which can help to prevent or treat some digestion-related conditions [25]. It also acts as a prebiotic, promoting the growth of beneficial gut bacteria [26].

Bran is also a major source of phenolic acids that include derivatives of hydroxybenzoic and hydroxycinnamic acids that are being recognized as important contributors to the reported protective effect of wholegrain cereal consumption against chronic diseases. Phenolic acids, mostly present in the three layers of bran (pericarp, testa, and aleurone), are the most abundant phenolic compounds in wholegrain cereals [27]. These phenolics are present in free and bound forms esterified to cell walls [28,29,30], being the latter forms dominant in wholegrain cereals, except for rice that contains high levels of free compared with bound forms [29,31,32].

Ferulic acid (FA) is the most abundant phenolic acid in grains [27,29] accounting for nearly 90% and 75% of total polyphenols for wheat and oat, respectively [33,34,35]. FA dimers (DFA) are also present in the bran of grains crosslinking polysaccharides and proteins in the cell walls [36,37]. Studies have shown that DFA can be released from cereals by microbial enzymes in the colon being further metabolized and bioaccesible to enter the circulatory system [38].

In the present study, it is proposed the evaluation of the effect of grain choice (wheat vs. oat) and milling fraction (whole grain vs. bran) on the phenolic profile (free vs. bound phenolics), antioxidant profile (evaluated through different methodologies), nutrient profile, and glycemic index. The aim of this study was to characterize and evaluate the nutritional and antioxidant properties of wheat/oat grains and bran, in order to increase the knowledge of the potential bioactivity of these products and byproducts, facilitate the selection of those antioxidant capacity assays which better reflect the differences in their bioactive properties, and to promote the use of wheat and oat grains and bran as nutraceutical food ingredients.

## 2. Materials and Methods

### 2.1. Chemicals

2,2′-Azinobis 3-ethylbenzothiazoline-6-sulfonic acid (ABTS), 2,2′-diazobis-(2-aminodinopropane)-dihydrochloride (AAPH), Fluorescein, 2,2-diphenyl-1-picrylhydrazyl (DPPH), Folin–Ciocalteu (FC) reagent, gallic acid (GA), 6-hydroxy-2,5,7,8-tetramethyl-2-carboxylic acid (Trolox), and ferulic acid (FA), pancreatin (P-1750), amyloglucosidase (A-9913), and amylase (A-3176) were obtained from Sigma-Aldrich, Co. (St. Louis, MO, USA). Amyloglucosidase enzyme (EC 3.2.1.3) and glucose oxidase–peroxidase kit (GOPOD) were purchased from Megazyme International Ireland (Wicklow, Ireland). All the solvents were HPLC grade (Sigma Aldrich Co., Madrid, Spain and Merck KGaA, Darmstadt, Germany).

### 2.2. Raw Materials

**Figure 1:**
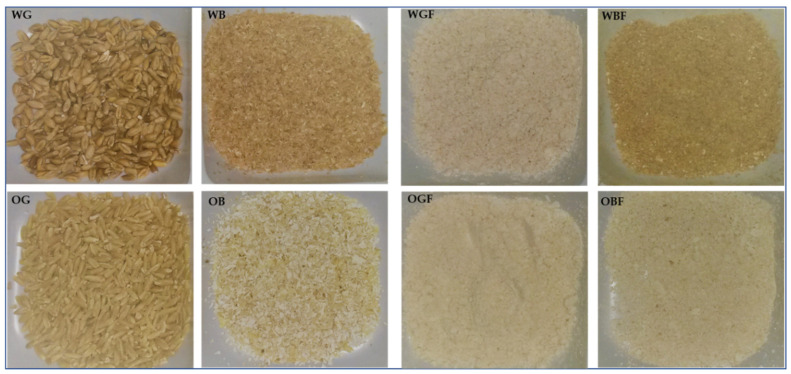
Raw material used for analysis. Wheat grain (WG), wheat bran (WB), oat grain (OG) and oat bran (OB) and the flours milled for all the samples: Wheat grain flour (WGF), wheat bran flour (WBF), oat grain flour (OGF), and oat bran flour (OBF).

Wheat (WG) and oat (OG) grain and their brans were kindly supplied by Emilio Esteban, S.A. (EMESA, Valladolid, Spain). Whole wheat grains (*Triticum aestivum*, cv. *Berdún*) grown in La Mudarra (Valladolid, Spain) and oat grain (*Avena sativa*, cv. *Ivory*) grown in Burgos, Spain, both harvested at grain full ripening stage during the growing season 2018. Wheat bran (WB) and oat bran (OB) were produced and provided from same supplier and both brans showed a proportion of particle sizes between 1000 and 2000 μm. All the samples were milled (Figure 1) into fine powder with a refrigerated blender IKA M 20 (IKA-Werke GmbH & Co. KG, Staufen im Breisgau, Germany) and samples were stored in air-tight polyethylene/plastic bags in dark conditions at room temperature until further analyses.

### 2.3. Proximate Composition

The moisture contents of grain and bran flours were assessed by drying 5 ± 0.001 g of powdered sample at 105 °C for 3 h. Total protein content was determined by the Dumas method (AOAC 2005, method 990.03) [39] in an elemental analyzer. A conversion factor of 5.7 was used to convert nitrogen into protein values. Total fat content was determined using dried samples extracted with petroleum ether (BP 40–60 °C) during 4 h in a Soxtec fat extracting unit (AOAC 2005, method 2003.05) [39]. Carbohydrates were estimated by difference. Total dietary fiber content was evaluated using the kit provided by Sigma (St. Louis, MO, USA), according to the manufacturer’s instructions, based on AOAC method 985.29 [39]. Ash content was determined by sample incineration in a muffle furnace at 550 °C for 5 h (AOAC 2005, method 923.03) [39]. All results were corrected for moisture content and expressed as g 100 g^−1^ of dry matter (d.m.).

For analysis of total dietary fiber, the samples were submitted to simulated physiological conditions of human digestion, according to the method proposed by Goñi et al. [40], which includes enzymatic treatments with porcine pepsin (0.2 mL of a 300 mg/mL solution in HCl-KCl 0.2 M buffer, pH 1.5, 40 °C, 1 h; Sigma-Aldrich), pancreatin (1 mL of 5 mg/mL solution in phosphate buffer 0.1 M; pH 7.5, 37 °C, 6 h), and pancreatic α-amylase (1 mL of a 120 mg/mL solution in Tris maleate buffer 0.1 M; pH 6.9, 37 °C, 16 h). After the enzymatic hydrolysis, the samples were centrifuged (15 min, 25 °C, 8000× *g*). The residues were considered as the insoluble dietary fiber (IDF) and supernatants were collected for determination of soluble dietary fiber (SDF). IDF residues were sequentially hydrolyzed with 12 M (1 h, 30 °C) and 1 M (1.5 h, 100 °C) sulfuric acid to yield soluble monomer constituents that were measured after reaction with dinitrosalicylic acid and insoluble residue quantified gravimetrically as Klason lignin (KL) after a drying process at 105 °C to constant weight.

For determination of SDF, supernatants were incubated (45 min, 60 °C) with 100 µL amyloglucosidase (Sigma-Aldrich), transferred into dialysis tubes (12,400 Da molecular weight cut off) and dialyzed against water for 48 h at 25 °C (water flow 7 L/h). An aliquot of dialyzed supernatants was hydrolyzed with 1 M sulfuric acid (100 °C for 90 min) for estimating SDF content after reaction with dinitrosalicylic acid. Total dietary fiber (TDF) was quantified as the sum of IDF components (non-starch polysaccharides (NSP) and Klason lignin) plus SDF, and results were expressed as g/100 g of d.m.

### 2.4. Fatty Acid Profile and α-Tocopherol Content

#### 2.4.1. Fatty Acid Profile

It was determined for all grains and bran flours. Lipids were extracted according to the method of Bligh and Dyer [41]. Lipid-containing chloroform phase was separated and evaporated. The remaining phase was dissolved in 1 mL of hexane, and a methylated procedure was carried out by adding 100 μL of 0.5 M methanolic KOH and leaving the reaction for 10 min at room temperature. The upper layer was transferred to a 2 mL vial. Analysis of fatty acid methyl esters (FAME) was carried out on a gas chromatograph Agilent 7890A (Agilent Technologies, Santa Clara, CA, USA) with a flame ionization detector. A DB-23 column 60 m × 0.32 mm, (0.25 μm film thickness). Helium was used as the carrier gas. The oven temperature was programmed to 50 °C for the first 7 min and increased up to 200 °C at a rate of 25 °C per min, then the temperature was further increased to 230 °C at a rate of 3 °C per min and held for 26 min. Injection and detector temperatures were 250 °C and 280 °C, respectively. One microliter of the hexane extract was injected in split mode (ratio 25:1), and FAME’s were identified by comparison of retention times with those of the standard (37 FAME’s mix, Supelco, Sigma-Aldrich). Results were expressed as percentage of total fatty acids.

#### 2.4.2. α-Tocopherol Content

It was determined according to the AOCS official method [42], using an Agilent 1200 series HPLC (Agilent Technologies, Palo Alto, CA, USA) equipped with a diode array detector. One milliliter of Bligh and Dyer [41] extract was evaporated under nitrogen and redissolved in 0.5 mL of hexane with tocopherol acetate as internal standard. Next, 140 µL of each sample and 10 µL of tocopherol acetate as internal standard was used. Then, an aliquot of 20 µL was injected in a normal phase column (250 mm × 4.6 mm, 5 µm) (Teknokroma Analítica S.A, Barcelona, Spain) to 30 °C. Elution was performed with an isocratic mixture of hexane/2-propanol (99.6:0.4; *v*/*v*) at a flow rate of 1.2 mL min^−1^ for a total run of 10 min. Detection was carried out using a UV detector at 292 nm and 284 nm for tocopherol acetate. Results were expressed in µg tocopherol per g of sample.

### 2.5. Phenolic Extracts Preparation

Free and bound phenolic compounds were extracted from grains (wheat and oat) and brans (wheat and oat).

#### 2.5.1. Extraction of Free Phenolic Compounds (FP)

One gram of each milled sample was weighed and mixed with 20 mL of chilled EtOH/H_2_O (80:20, *v*/*v*). Extraction was carried out by magnetic stirring for 10 min at room temperature. After centrifugation (25 °C, 2500× *g*, 10 min), the supernatant was collected and filtered through Whatman’s No. 1 paper. The pellet was further extracted repeating the previous step. Both supernatants were pooled, evaporated to dryness using a rotary evaporator in vacuum (30 °C), dried under a gentle flow of nitrogen gas, and redissolved in 10 mL of MeOH/H_2_O (80:20, *v*/*v*). The extracts were filtered through a nylon filter (0.22 µm, 25 mm) and stored at −80 °C until further analysis.

#### 2.5.2. Extraction of Bound Phenolic Compounds (BP)

The residue from the free phenolic compounds extraction was subjected to alkaline and acid hydrolysis to recover the bound phenolic compounds. Then, 12 mL of distilled water and 5 mL of 10 M NaOH were added to the residue and stirred overnight at room temperature using a magnetic stirrer. The pH of the solution was adjusted to pH 2, and released phenolic acids were re-extracted three times using 15 mL of ethyl acetate and centrifugated (25 °C, 12.857× *g*, 10 min). The organic layers were combined and refrigerated.

After alkaline hydrolysis was completed, acidic hydrolysis was performed by adding 2.5 mL of concentrated HCl and incubating in a water bath at 85 °C for 30 min. The sample was then cooled down and phenolic compounds extraction using ethyl acetate was performed in the same manner as described above for alkaline hydrolysates. Both organic fractions obtained from alkaline and acid hydrolysis were mixed, evaporated at 30 °C to dryness using a rotary evaporator and redissolved in 10 mL of MeOH. The bound phenolic extracts were filtered through a Nylon filter (0.22 µm, 25 mm) and stored at −80 °C until further analysis.

### 2.6. Total Phenols (TP) Quantification

TP were measured in the free and bound fractions using the Folin–Ciocalteu reagent, according to Slinkard and Singleton [43], with modifications [44]. The absorbance was measured at 765 nm using a microplate reader (Fluostar Omega, BMG, Ortenberg, Germany). Results were expressed as mg of gallic acid equivalents (GAE) 100 g^−1^ d.m.

### 2.7. Determination of Free and Bound Phenolic Compounds Using HPLC-DAD-ESI/MS and HPLC-DAD

Soluble and insoluble phenolic compounds were analyzed following the method described previously by García-Villalva et al. [45]. Analysis of phenolic compounds was performed using 1100 series high performance liquid chromatograph (HPLC) coupled to a photodiode array detector (model G1315B, Agilent, Santa Clara, CA, USA). The equipment was controlled using the Agilent software v. A.08.03 (Santa Clara, CA, USA). Sample injection volume was 20 μL and compounds were retained in a C_18_ Kinetex-F5 column (5 μm, 250 × 4.6 mm; Phenomenex, Macclesfield, UK) and eluted using 1% formic acid in water (mobile phase A) and acetonitrile (mobile phase B) at a flow rate of 1 mL/min and the following gradient program: from 5% B to 60% B in 37 min, from 60% B to 98% B in 3 min, from 98% B to 5% B in 5 min. The chromatograph was connected to an ion-trap mass spectrometer with an electrospray interface (Agilent, Santa Clara, CA, USA). Mass spectrometry was performed using a negative ion mode and a mass scan range from 100 to 1200 m/z. Mass spectrometry analysis parameters used were flow rate of nitrogen of 11 L/min, nebulizer gas pressure of 65.0 psi, voltage of 3.5 kV and capillary temperature of 350 °C. For collision-induced fragmentation in the ion trap helium and a collision energy of 50% were applied. The phenolic compounds were identified according to their maximum absorbance at wavelengths 290 and 320 nm, molecular mass, and fragmentation pattern. Representative examples of the chromatographic profile of wheat and oat soluble and insoluble extracts at 290 nm and 320 nm have been included as Supplementary Figure S1. Quantification was made by external calibration curves in the concentration range between 1 and 100 µg mL-^1^ of standard compounds (Sigma-Aldrich, Madrid, Spain): FA (*y* = 2.71 × 10^6^x + 1.53 × 10^6^; R^2^ = 1.00), caffeic acid (*y* = 51.33 × 10^6^x + 11.00 × 10^6^; R^2^ = 0.99), and apigenin 8-*C*-glucoside (*y* = 9.30 × 10^6^x + 5.28 × 10^5^; R^2^ = 1.00). Analyses were performed in duplicate and results were reported as in (μg g^−1^ d.m.).

### 2.8. Total Antioxidant Capacity (TAC)

TAC was measured on the free and bound phenolic fractions using 2,2-diphenyl-1-picrylhydrazyl (DPPH) radical scavenging activity, oxygen radical absorbance capacity (ORAC), 2,2′-azinobis-(3-ethylbenzothiazoline-6-sulfonate (ABTS) methods, and ferric reducing ability of plasma (FRAP).

#### 2.8.1. DPPH Assay

The antioxidant activity of the extracts against the DPPH radical was estimated according to the procedure described by Brand-Williams et al. [46] with modifications. An amount of 100 µL of extracts was mixed with 400 µL of MilliQ water and 500 µL of DPPH working solution (120 µM using methanol as solvent) in a 96-well microplate. Absorbance at 515 nm was recorded for 30 min in a microplate reader (Fluostar Omega, BMG Ortenberg, Germany). Trolox was used as the standard (7.5–240 µM). The results were expressed as µmol Trolox equivalents (TE) 100 g^−1^ d.m.

#### 2.8.2. ORAC Assay

The procedure was based on a previously reported method with slight modifications [47]. The standard curve of Trolox (7.5–240 µM) and samples were diluted in phosphate buffer (75 mM, pH 7.4). A volume of 150 μL fluorescein was placed in 96-well black polystyrene plates, and 25 μL of Trolox standard, sample or phosphate buffer as blank were added, all in duplicates. Samples, standards, and blanks were incubated with fluorescein at 37 °C for 3 min before 2,2′-azobis (2-methyl propionamidine) dihydrochloride solution was added to initiate the oxidation reaction. Fluorescence was monitored over 100 min with a microplate reader (Fluostar Omega, BMG Ortenberg, Germany), using 485 nm excitation and 528 nm emission filters. Results were calculated using the areas under the fluorescein decay curves, between the blank and the sample, and expressed as µmol TE 100 g^−1^ d.m.

#### 2.8.3. ABTS Assay

Antioxidant capacity against diammonium salt of ABTS radical was evaluated following the method first described by Re et al. [48], as modified by Martin-Diana et al. [44]. Then, 100 µL of diluted samples was mixed with 1000 µL ABTS·^+^ working solution in an Eppendorf tube. The decay in absorbance at 734 nm was recorded over 30 min with a microplate reader. Trolox was used as the standard (7.5–240 µM). The absorbance was measured at 734 nm with a microplate reader (Fluostar Omega, BMG Ortenberg, Germany). Results were corrected for moisture and expressed as as µmol TE 100 g^−1^ d.m.

FRAP assay was performed following the protocol reported by Pereira et al. [49]. Absorbance at 700 nm was recorded in a microplate reader. FeSO_4_·7H_2_O was used as the standard (4.0–2.2 mM). The results were expressed as µmol Fe equivalents 100 g^−1^ d.m.

#### 2.8.4. Relative Antioxidant Capacity Index (RACI)

The Relative antioxidant capacity (RACI) of the samples was used as an integral concept which allows the comparison of antioxidant capacity derived from different chemical methods [50]. RACI values were determined through the following equation: (x − µ)/σ, where x is the antioxidant value, µ is the average value of the results of the corresponding method (DPPH, ORAC, ABTS, or FRAP), and σ is the standard deviation.

### 2.9. Glycemic Index (GI)

The GI was determined in the brans, first, measuring the content of available starch using the total starch assay kit of Megazyme (Bray, Ireland). Then, the in vitro starch hydrolysis rate was determined as described by Gularte and Rosell [51]. Glucose analysis was performed using a GOPOD kit (Megazyme, Bray, Ireland). Hydrolysis index (HI) and glycemic index (GI) values were calculated as proposed by Granfeldt et al. [52].

### 2.10. Color Analysis

Color was measured using a colorimeter (CM-2600d, Konica Minolta Osaka, Japan) with the D65 standard illuminate, 45/0 sensor and the 10° standard observer. The instrument was calibrated with a while tile standard (L* = 93.97, a* = −0.88 y b* = 1.21). The color parameters; lightness (L*), redness (a*) and yellowness (b*) were expressed in accordance with the CIELab color space. Measurements were taken on the samples packaged in transparent plastic bags at ten different points per sample. To reduce plastic reflection, the specular component was excluded (SCE mode).

### 2.11. Statistical Analysis

Data were analyzed by one-way analysis of variance (ANOVA) with Statgraphics Centurion XVI software. Fisher LSD (least significant difference) test was applied for determining group differences at 95% significance level. Principal component analysis (PCA) were performed on standardized data to elucidate the relationships among variables (FA and bioactivity of parameters).

## 3. Results and Discussion

### 3.1. Proximate Composition

Proximal composition (ash, fat, moisture, protein, carbohydrates, and soluble and insoluble fiber) was analyzed in WG, WB, OG, and OB (Table 1). Ash content in both grains was close to 2%, whereas a large difference was observed between the brans, where WB showed the highest ash content (7%) followed by OB (3%). Fat content was significantly higher in OG and OB (9.20 and 7.27 g 100 g^−1^, respectively) compared to WG and WB (2.18 and 2.73 g 100 g^−1^, respectively). In the case of WG protein content was 9.72 g 100 g^−1^. Since protein content is a characteristic of wheat grain classification, the cultivar used in the study could be considered as a low protein wheat type. OG protein content was slightly higher than WG (10.49 g 100 g^−1^) and similar to the reported values for husked oat [53].

Carbohydrate content was close to 76% (75.94 g 100 g^−1^) in WG, of which nearly 15% (15.10 g 100 g^−1^) were fiber. Compared to WG, OG were characterized by a lower carbohydrate (60.12 g 100 g^−1^) and fiber (11.72 g 100 g^−1^) contents that were in the range reported in the literature for husk oat [53,54]. The ash content of WG was 1.78 g 100 g^−1^ and 1.69 g 100 g^−1^ in the case of OG. Moisture was higher in WG than OG ranged both values between (8.92 to 9.45).

OG showed almost double value of SDF (3.06 g 100 g^−1^) than WG (1.35 g 100 g^−1^); meanwhile, in WG, IDF formed by NSPs and KL was higher. IDF in cereals consists mainly of lignin, cellulose, and hemicellulose, compounds that are responsible for the rigid structure of cell walls. Once in the digestive tract, the insoluble fiber is not broken down, maintaining the amount of fiber mass, increasing water retention and stimulating regular bowel movements. Lignin is also considered important from a functional point of view since it is thought to act as a physical barrier to microbes, reducing their fermentation capacity [55].

WB and OB nutritional composition varied as reported in the literature, where ranges of 15–18% protein, 10–50% starch and sugars, 5–10% fat, 10–40% total dietary fibers were found [56,57]. The WB showed a protein content significantly lower than that found in OB. Values of total fiber were twice higher for WB compared to OB (53.89 vs. 24.18 g 100 g^−1^), although SDF in OB (4.5%) were higher than WB. The IDF was higher in WB although lignin content of this fraction was higher in OB.

### 3.2. Fatty Acid Profile (FA) and α-Tocopherol Content

**Figure 2:**
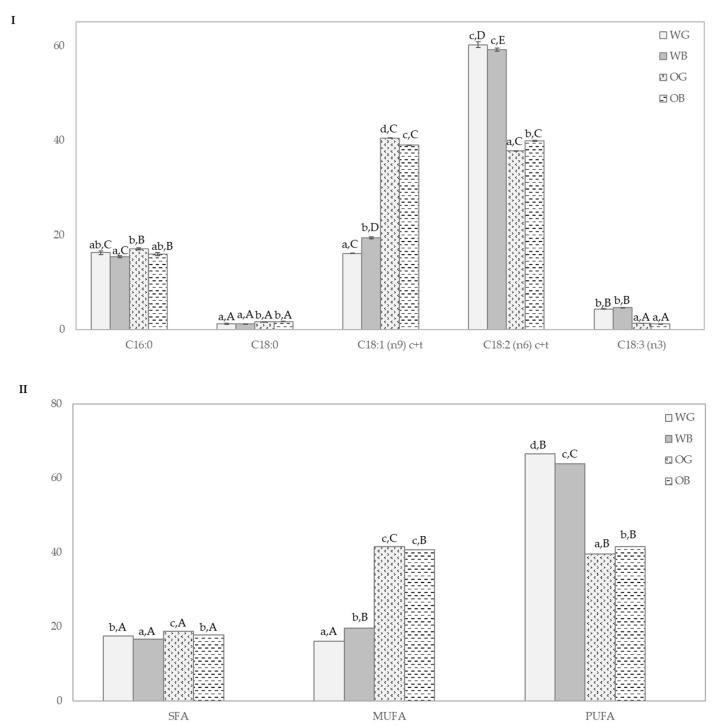
Fatty acid profile of wheat grain (WG), oat grain (OG), wheat bran (WB), and oat bran (OB). (**I**) More abundant fatty acids: palmitic (C:16:0), stearic (C:18:0), oleic (C:18:1(n9)), linoleic (C:18:2 (n6)), and α-linolenic acids (C:18:3 (n3)) and (**II**) Fatty acid groups: saturated fatty acids (SFA), monounsaturated fatty acids (MUFA) and polyunsaturated fatty acids (PUFA). Results were expressed as % of total fatty acids. Different lowercase letters indicate significant differences (*p* < 0.05) in a given fatty acid among samples (WG, WB, OG, and OB) and capital letters indicate significant differences (*p* < 0.05) in a given sample (WG, WB, OG, or OB) among fatty acids (*p* < 0.05).

The fatty acid composition expressed as % of total fatty acids is shown in Figure 2. All the samples showed a similar fatty acid profile (Figure 2I), being unsaturated fatty acids linoleic (C:18:2 (n6)), oleic (C:18:1(n9)), and palmitic acids (C:16:0) the most abundant compounds accounting for 90–95% of total fatty acid content in agreement with the literature [58]. In OG and OB, essential fatty acids such as linoleic and α-linolenic reached up to 75% of the total content.

In WG and WB linoleic acid was the most abundant compound of the fatty acids fraction accounting for 58–62% of total content followed by oleic acid (18–21% of total content) whereas palmitic acid (C:16:0), linolenic (C:18:3), and stearic acids (C:18:0) were minor compounds of the fatty acid fraction. The WG and WB showed significant higher values of linoleic acid compared to OG and OB, meanwhile oleic acid showed an opposite trend with double concentration in OG and OB. The results observed were in accordance with other published studies [59]. As expected, polyunsaturated fatty acids were preponderant over saturated and monounsaturated fatty acids in all samples (Figure 2II).

Alpha-tocopherol content was evaluated in all samples (Supplementary Figure S2). The results showed approximately double α-tocopherol amount in grains, as compared with brans. These results are in accordance with previous work, as tocopherols has been found in higher proportion in wholegrain flours than in refined counterparts, and particularly located in the germ fraction [60,61,62].

### 3.3. Determination of Free and Bound Phenolic Content and Composition

**Figure 3:**
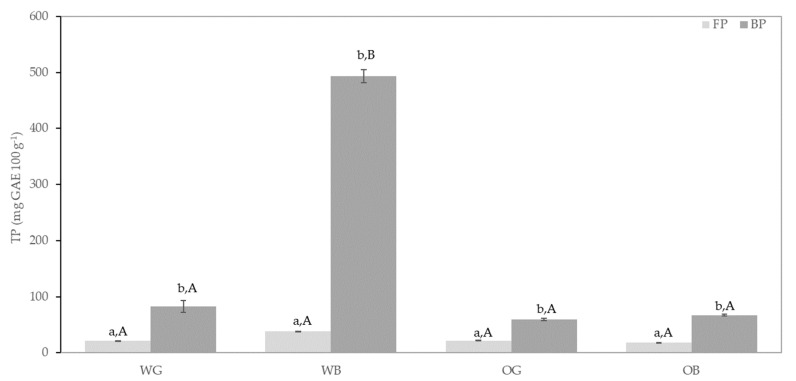
Total Phenolic compounds (TP) content for free phenolic fraction (FP) and insoluble phenolic fraction (BP) of wheat grain (WG), oat grain (OG), and their bran, wheat bran (WB) and oat bran (OB). Results were expressed mg GAE/100 g of dry sample. Different lowercase letters indicate significant differences (*p* < 0.05) in a given sample (WG, WB, OG, or OB) between fractions (FP vs. BP) and capital letters indicate significant differences (*p* < 0.05) in a given fraction (FP or BP) among samples (WG, WB, OG, and OB).

Total content of FP and BP was determined using the Folin–Ciocalteu method and expressed as mg of gallic acid equivalent (GAE) per100 g of grain (Figure 3). Results showed higher amounts of BP than FP in all the samples. According to the literature, there is a higher contribution of BP over FP to the total phenolic fraction in either the wholegrain or bran of most of cereals [63]. Interestingly, separation of bran from grain kernel resulted in a dramatic increase in the total content of FP and BP, which was more evident in wheat compared to oat. Similarly, Liyana-Pathirana and Sahidi [64] reported approximately double and triple amounts of total FP and BP, respectively, in WG compared to WB. Among studied samples WB was the best source of FP and BP whereas much lower values were found in WG, OG, and OB characterized by similar FP and close BP amounts.

Composition of FP and BP extracts for wholegrains and brans of wheat and oat is shown in Table 2. Tentative characterization of phenolic compounds, free and bound, were performed from accurate mass measurements of parent and fragment ions and comparison with literature data [65].

Sum of identified compounds in the soluble phenolic extracts indicated that OG was a better source of FP (194.6 μg g^−1^) compared to OB (208.2 μg g^−1^). In addition, oat raw materials were characterized by lower FP levels compared to wheat samples (283.9 and 853.9 μg g^−1^), with WB being the better source of FP. Sum of identified compounds in BP fraction indicated that WB was the richest source of BP (19.4 mg g^−1^) with amounts over three and ten times higher than the observed for WG (4.7 mg g^−1^) and oat samples (1.6 and 2.1 mg g^−1^ OG and OB, respectively).

The determination of individual FP content evidenced high variability among the investigated wheat and oat samples. Regarding WG, phenolic acid class was represented by FA, DFA and caffeic acid glucosides, with the former being the most abundant compound (76.4% of total phenolic content) followed by DFA (13.7% of the total phenolic content) mainly distributed in the BP fraction. Apigenin *C*-glycoside, diglycosides, and triglycoside, mainly segregated in the FP fraction, were the representatives of the flavonoid class in WG, with vitexin (apigenin-8-*C*-glycoside) as the major compound. Lignans such medioresinol were identified in the BP fraction being a minor phenolic compound in WG. Phenolic profile of WB characterized by higher abundance of phenolic acids (92% of total phenolic content mainly composed of FA and DFA), followed by lignans (4.6% of total phenolic content) and apigenin glycosides (3.31% of total phenolic content), was similar to the observed for WG although some differences become apparent. First, amounts of free and bound FA, caffeic 4-glucoside, vitexin, apigenin-6-*C*-galactosyl-8-*C*-glucosyl-*O*-glucuropyranoside, medioresinol, DFA II and III were much higher than the found in WG. Moreover, the total content and diversity of apigenin diglycosides in WB was lower compared to WG and vicenin-2, 1-acetoxy pinoresinol and daizdein were exclusively identified in WB. Phenolic profile of OG was made up of phenolic acids (81.94% of total content) including in order of abundance FA, DFA and caffeic 4-glucoside as the main phenolic group, with the two former mostly or uniquely found as bound forms, respectively. According to their abundance, flavonoids (4.95%), aventhramides (4.64%), and lignans were minor phenolic groups in OG. The flavonoid class was represented by free flavones such as apigenin glycosides, daidzein and glycosilated/acetylated-3′,4′,5′-trihydroxy-3,7-dimethylflavone. Aventhramides K, 2c, 2p and 2f were identified in OG, being the second compound the predominant. Bound medioresinol was the only lignan identified in OG. Phenolic distribution in OB was similar to the observed for OG being phenolic acids the predominant group followed by flavones, aventhramides, and lignans. Slightly higher amounds of caffeic 4-glucoside, apigenin-6/8-C-pentoside-8/6-C-hexoside I, vitexin, aventhramide K and DFA I were detected what suggest a higher distribution of these compounds in the bran. In contrast, most aventhramides forms, glycosilated/acetylated-3′,4′,5′-trihydroxy-3,7-dimethylflavone, medioresinol and DFA III were lower in the bran as compared to OG.

In the literature, earlier studies reported a lower range of presence of caffeic acid (0.9–16.8 μg g^−1^) than the results obtained in this work [66]. Calinoiu et al. [67] reported apigenin-glucoside (20.40 μg g^−1^) in wheat bran samples; although, this group of phenolic compounds were not found in oat bran, but similar levels to those found in this work of avenanthramides were reported (4.14–7.85 μg g^−1^). Shewry et al. [68], as published in a multi-variety work with OG detected avenanthramides (12.7–44.9 μg g^−1^) in the range of the results observed for OG and OB samples. The free phenolics found in this study are slightly lower to the levels found by Verma et al. [69], who evaluated the phenolic content in the bran fraction of a wide range of wheat varieties. The BP, on the other hand, showed a range within that reported in Verma et al. [69]. These authors found a range of 854 to 1754 μg g^−1^ for free phenolics, and after saponification and solvent extraction a range of 3406 to 6702 μg g^−1^ for bound phenolics.

Ferulic acid (Table 2) is one of the most ubiquitous phenolic compounds found in nature [70] and was the most abundant in the insoluble fraction. The levels found are in the range of those previously reported for wheat and oat whole grains [70]. According to previous studies, the ferulic acid levels found may provide functionality against inflammation, at least in WB, but it would have a tenfold lower concentration in the rest of the samples [71]. The levels of ferulic acid in the soluble fractions were very low in the case of wheat, as expected, and FA was not detected in the case of oat soluble fractions. The level of ferulic acid found in WG samples may provide a significant in vivo antioxidant effect in plasma, according to Price et al. [72], who demonstrated 1200 µmol dietary dose of FA (eq. to 232 mg), significantly increased the ferric reducing ability of plasma. Previous intervention results in human have shown that WG may effectively serve as functional ingredient. A 67 g WG dose would provide the required ferulic acid level, which is a suitable amount. On the other hand, a bran portion of 9 g of WG would provide a similar level of ferulic acid (31.23 mg) as that used by Price et al. (33.8–37.4 mg) [71], where a significant effect on inflammatory was observed.

### 3.4. Total Antioxidant Capacity (TAC)

The antioxidant activity was evaluated through different methodologies, in order to better approach the characterization of the total antioxidant capacity (TAC) of the samples [50].

The results obtained for antiradical activity against DPPH show a similar trend as observed for results on total phenolic compounds (Figure 3). The antiradical capacity of the bran samples increased over those of the whole grain, with a higher antiradical capacity of the bound fractions over the soluble ones. Previous work has shown the DPPH methodology not totally correlated with total phenolic values in wheat and oat bran samples [67]. Calinoiu et al. [67] found higher DPPH inhibitory activity in oat bran than in wheat bran, despite a lower total phenolic content in the former, as compared with the wheat bran. In the present work, a better correlation of the two methodologies, TPC and DPPH, can be observed (Table S1).

**Figure 4:**
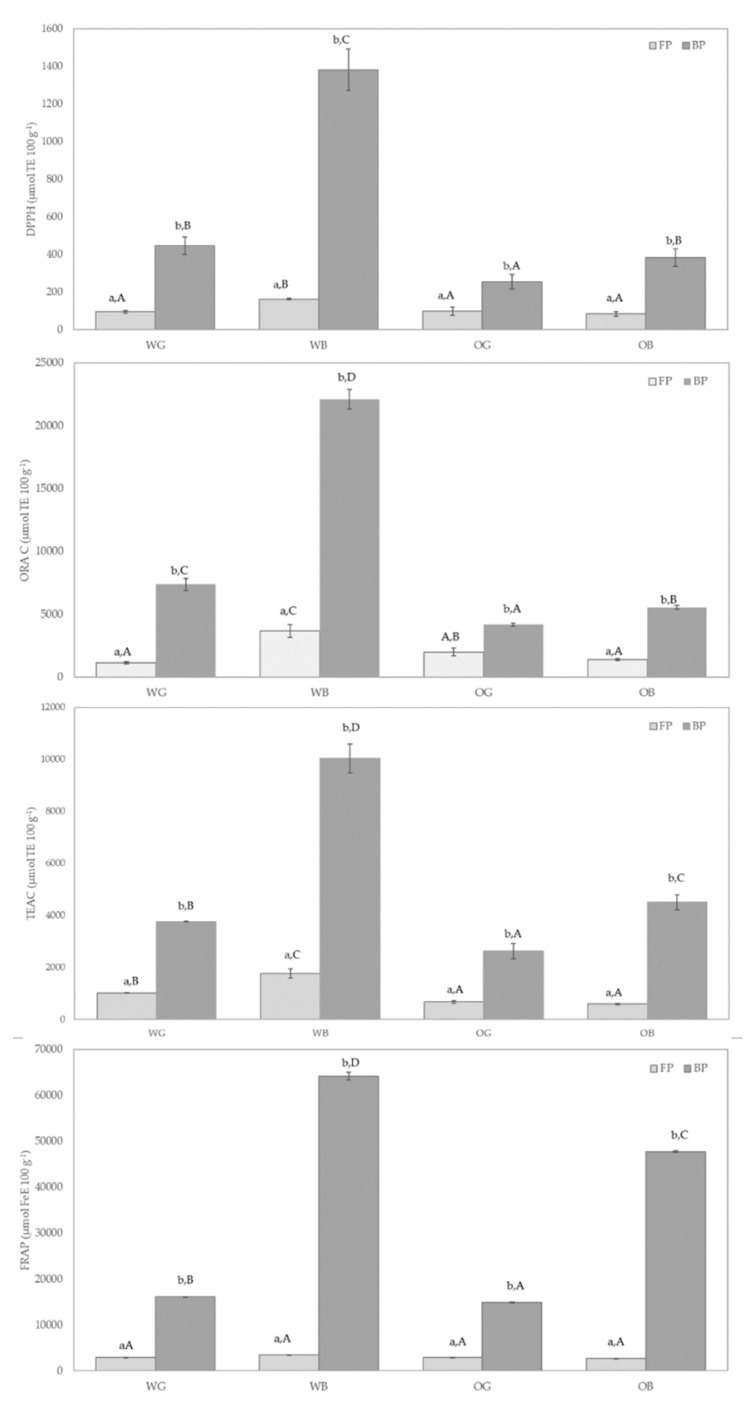
DPPH, ORAC, ABTS, and FRAP values for free phenolic fraction (FP) and insoluble phenolic fraction (BP) of wheat grain (WG), oat grain (OG), and their bran, wheat bran (WB) and oat bran (OB). Results were expressed in µmol TE 100 g^−1^ for ORAC, DPPH, ABTS, and µmol Fe E 100 g^−1^ for FRAP of dry sample. Different lowercase letters indicate significant differences (*p* < 0.05) in a given sample (WG, WB, OG, or OB) between fractions (FP vs. BP) and capital letters indicate significant differences (*p* < 0.05) in a given fraction (FP or BP) among samples (WG, WB, OG, and OB).

A good correlation was also obtained between ORAC and TP and DPPH values (Supplementary Table S1, Figure 4). The ORAC methodology evaluates the antioxidant activity against peroxyl radicals [73], and in this regard, it is generally considered as indicator of a protective effect against not only rancidity in food systems, but also preventing oxidative damage in the human body [74]. ORAC values of the brans, particularly for the bound fractions, were more than threefold higher for wheat than for oat, while these differences were not so marked with the rest of the antioxidant results. This may imply a more efficient protection against lipid oxidation from food formulations including wheat bran, over those containing oat bran. The results obtained with the FRAP method (Figure 4) resulted in the lower correlation with the rest of the antioxidant assays. The capacity of the samples to reduce iron and to potentially provide protection against hydroxyl radical formation is evaluated in this method [75]. In this regard, the differences between wheat and oat were no significant, except for the bound fractions, which resulted in a significantly (*p* < 0.05) higher FRAP activity of the wheat bran over the oat bran, although this difference was to a much lower extent than those observed with the rest of the antioxidant methodologies.

Significant differences were obtained from the different antioxidant capacity methodologies applied, and the wheat samples, either grain or bran, resulted in all cases with higher antioxidant capacity than those of oat. Similar results have been previously reported, with higher TP and antioxidant response of wheat over oat bran fractions [67]. Positive correlations of antioxidant markers such as TP, DPPH, and ABTS have been also previously observed in cereal-based samples [76,77]. Soong et al. [76] reported a significant direct relationship of TP with ABTS and DPPH-based antiradical activities. The differences observed in the antioxidant results between whole grain and bran, under the several methodologies tested, support a higher in vitro antioxidant capacity of the whole grain that that of the refined flours, which lack the bran fraction, as it has been reported previously [78].

**Figure 5:**
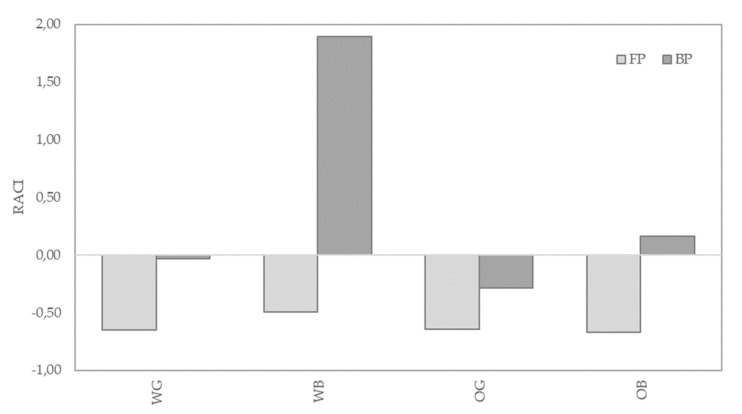
RACI values for free phenolic fraction (FP) and insoluble phenolic fraction (BP) of wheat grain (WG), oat grain (OG), and their bran, wheat bran (WB) and oat bran (OB).

The RACI index [50] was calculated as an integrated approach for the antioxidant capacity of the different samples (Figure 5). This tool may help to the comparison of different food and ingredients from data of works evaluating the antioxidant capacity through different methodologies. RACI is a numerical scale that can integrate multiple chemical methods, thus allowing the comparison of the antioxidant capacity in a large number of samples. The highest RACI index corresponded to the BP of WB, followed by BP of OB, WG, and OG. Following BP, the RACI of FP were ranked in the following order: WB, OG, WG, and OB. Correlation analysis also indicated a higher association of RACI with ORAC values of BP, and with FRAP values of FP (Table S1).

**Figure 6:**
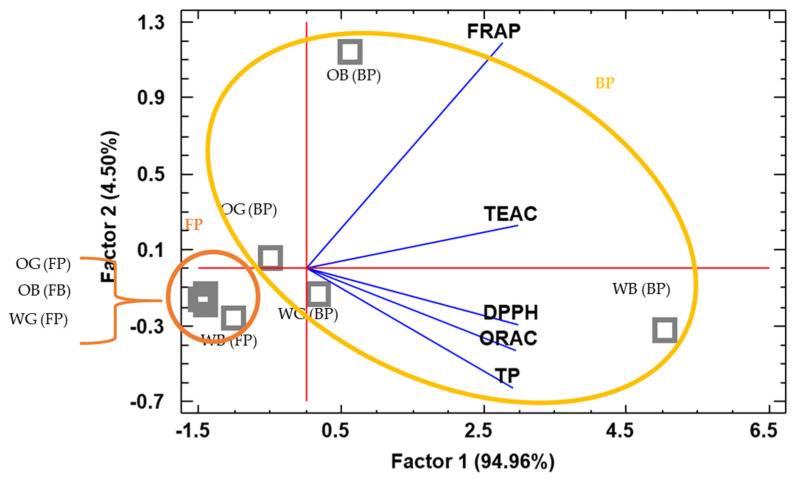
PCA analysis of wheat grain (WG), oat grain (OG), wheat bran (WB), and oat bran (OB). Orange and yellow circles group scores for free (FP) and bound (BP) phenolics, respectively.

The PCA analysis resulted in a separation of the samples based on antioxidant profile of FP and BP fractions (Figure 6). As indicated by the lower correlation of FRAP values (Supplementary Table S1), FRAP assay resulted in a markedly different contribution to Factor 1 and 2 in the PCA analysis than that provided by the rest of the antioxidant assays (Figure 5). The FRAP results would in this regard better discriminate FP and BP extracts, as it can be observed in Figure 6, the blue axis for FRAP values better separates BP and FP data (circled groups). TEAC and FRAP are methods based on probes with a similar redox potential [73], and although they are carried out under different pH conditions, this may explain a better correlation of FRAP with TEAC than with the rest of the antioxidant assays. On the other hand, DPPH, ORAC, and TP methods were highly correlated, and would be to some extent redundant, but any of them would better discriminate the antioxidant results of wheat against oat samples, except for the FP fraction of the WG, which was not separated from the FP fractions of oat (OG and OB).

### 3.5. Color Analysis

**Figure 7:**
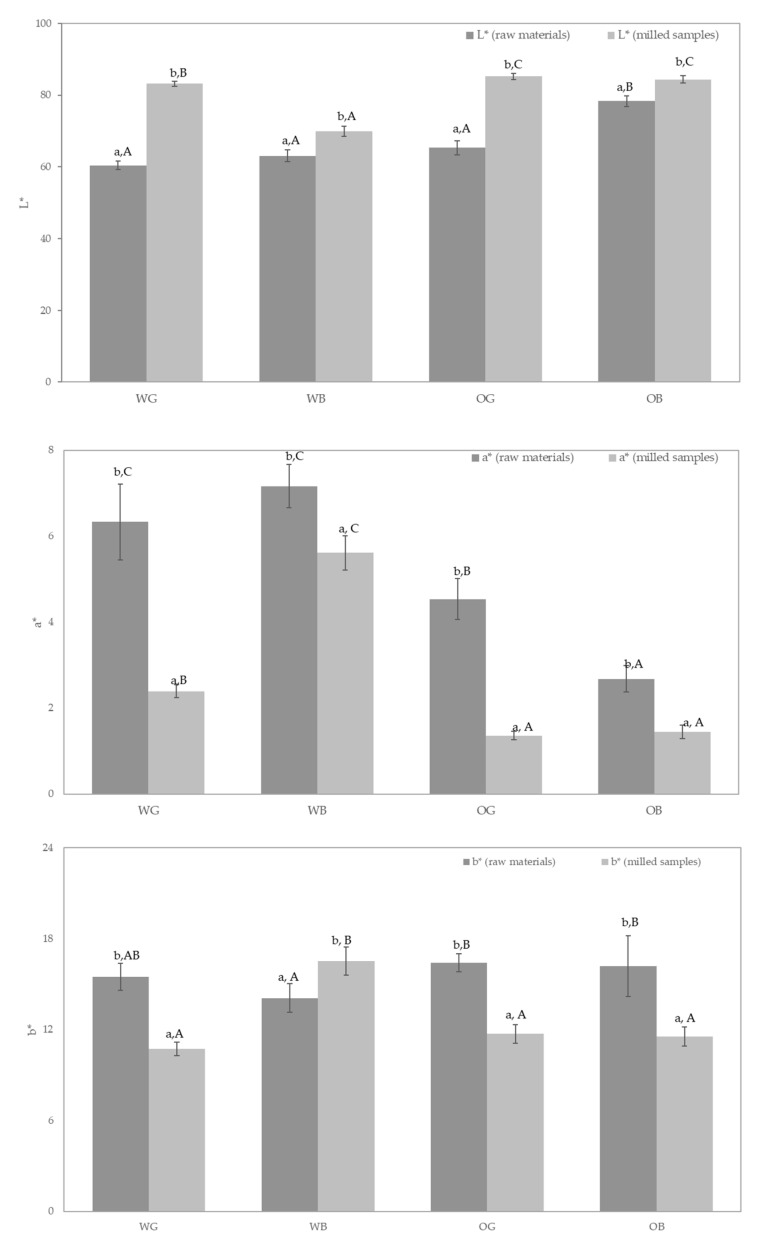
Colorimetric parameters (CIE L*a*b*) of wheat grain (WG), oat grain (OG), and their brans weight bran (WB) and oat bran (OB), as raw materials (dark grey) and milled flours (clear grey). Different lowercase letters indicate significant differences (*p* < 0.05) in a given sample (WG, WB, OG or OB) among raw or milled material and capital letters indicate significant differences (*p* < 0.05) in a given material (raw or milled) among samples (WG, WB, OG, and OB).

Color analysis was evaluated for all the samples (Figure 7). The results show a significantly higher luminosity (L*) of the OB samples, as compared to OG and wheat samples (WG and WB), previous to the milling process. As expected, milling increased L* of the samples, due to particle size reduction [79], but in a differently manner, depending on the type of sample. Bran samples resulted less affected by the milling process, and similar L* values were obtained in the WG and OG samples, as compared to final OB. WB resulted in the lowest luminosity for milled samples.

The a* parameter (green-red) values obtained for the different samples were in the positive range, indicating a red component, although to limited values. The wheat samples scored higher a* values, and the milling process reduced this component to values close to zero. As suggested by Yang et al. [80], these values may reflect anthocyanin content, and the heat produced during milling may have reduced its presence. In the case of b* parameter (blue-yellow), all showed similar values before milling, higher in range than the a* values, and regarding antioxidant activity, b* values are indicative of carotenoid content [81]. A similar behavior of the b* component after milling was observed, which resulted significantly reduced, with the exception of wheat bran, which showed increased b* values after milling.

### 3.6. Glycemic Index

**Figure 8:**
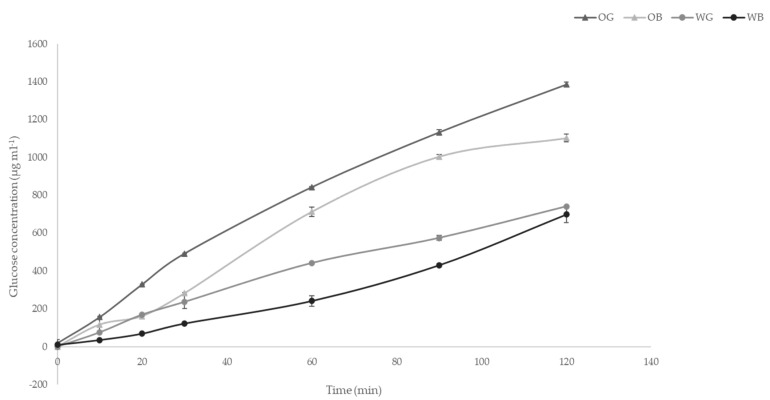
Kinetics of starch hydrolysis under in vitro conditions of wheat grain (WG), wheat bran (WB), oat grain (OG), and oat bran (OB) samples.

The Glycemic Index (GI) of the samples was evaluated through in vitro digestion kinetics (Figure 8). GI was calculated [52] from the area under the curve (AUC). GI results were 56.71, 58.53, 73.15, and 65.47 for wheat grain, wheat bran, oat grain, and oat bran, respectively. Wheat starch showed lower GI values than oat starch. In both cases, wheat and oat, the GI observed was reduced when evaluated in the bran fraction as compared to the whole grain. The GI was calculated as an intrinsic property of starch [82], and therefore the same amount of starch was used in all the assays. In this regard, the differences due to fraction used (grain vs. bran) would mostly be due to differences in the digestibility of the starch associated to the bran fraction, as compared to the most abundant in the endosperm. Moreover, physical interferences of the fiber with the enzymatic digestion of starch can be accounted in this type of in vitro assays, as it has been showed previously [83,84]. Xie et al. [85] showed significant differences in the starch isolated from wheat bran, as compared to the rest of starch in wheat. A lower amylose content in WB starch might explain the lower GI found [86], as compared to WG.

## 4. Discussion

Since nutraceuticals ingredients are dietary supplements made from natural bioactive substances found in food for health, and prevention and treatment of diseases, and their effects are based on the benefits of their nutrients, it is necessary to identify and quantify them in the raw material in order to predict the potential benefits for their whole or partial use of this material.

The results found confirmed the differences observed in the antioxidant properties between whole grain and bran, which support a higher in vitro antioxidant capacity of the whole grain than that of the refined flours, which lack the bran fraction and the interest to maintain or integrate bran part as ingredient.

Although bran has acquired increased importance over recent years for its potential benefits as part of human diet, OB is preferred over WB for its β-glucan content; however, this study shows the higher antioxidant activity of WB vs. OB which is one of the main properties of this byproduct.

Although different antioxidant parameters were evaluated, ferric reducing power (FRAP) results were those which better characterized differences between the two fractions (free and bound), which may be associated with differentiated bioactive properties of the bound phenolic compounds of both cereals, wheat, and oat. This characteristic may be of use for rapidly screening antioxidant-rich brans, as FRAP was the method that better correlated with the most abundant bound phenolic fraction, and also, it may be of help in future research in order to explain bioactivity mechanisms, as increased reducing power can inhibit radical formation in biological systems.

Ferulic acid content was significantly higher in the insoluble fraction and WB showed higher content than in OB either of the respective cereals, which makes this byproduct interesting from a nutraceutical point of view, associated to the bioactive properties reported for this phenolic acid. Ferulic acid and other phenolic compounds and derivatives have been proved to be effective antioxidant, anti-inflammatory, anti-diabetic, anti-cholesterolemic, i.e., other properties. Most of the diverse properties of FA may be associated with its ability of break free radical chain reactions and will explain the high antioxidant activity found in the study in those fraction rich in FA.

On the other hand, the study showed glycemic index of the starch present in the bran fractions of WB was low, as compared with that of wheat grain, and oat grain, and bran starches, which may give functional properties to WB-formulated products.

In conclusion, this study supports the significance of WB bioactive potential and may contribute to increase the interest in developing methodologies WB-based nutraceutical ingredients with increased bioavailability of bound phenolics.

## Figures and Tables

**Table 1:** Proximal composition of wheat grain (WG), wheat bran (WB), oat grain (OG), and oat bran (OB). Values were expressed as g 100 g^−1^ of sample. NSPs (non-starchy polysaccharides), KL (Klason Lignin).

		WG	WB	OG	OB
**Ash**		1.78 ± 0.03 ^b^	7.31 ± 0.04 ^d^	1.69 ± 0.02 ^a^	3.03 ± 0.01 ^c^
**Fat**		2.18 ± 0.01 ^a^	2.73 ± 0.46 ^b^	7.27 ± 0.26 ^c^	9.20 ± 0.78 ^d^
**Moisture**		9.45 ± 0.32 ^b^	13.64 ± 0.20 ^d^	8.92 ± 0.02 ^a^	10.02 ± 0.08 ^c^
**Protein**		10.66 ± 0.04 ^a^	13.19 ± 0.27 ^c^	11.50 ± 0.27 ^b^	17.63 ± 0.00 ^d^
**Carbohydrates**		75.94 ± 0.24 ^c^	63.14 ± 0.49 ^b^	70.63 ± 0.58 ^c^	60.12 ± 0.69 ^a^
**Soluble Fiber**	**NSPs**	1.35 ± 0.003 ^a^	1.55 ± 0.29 ^ab^	3.06 ± 0.34 ^b^	4.50 ± 0.33 ^c^
**Insoluble Fiber**	**NSPs**	11.56 ± 1.10 ^b^	43.18 ± 3.08 ^c^	7.17 ± 0.50 ^a^	7.71 ± 0.62 ^a^
	**Klason Lignin**	2.01 ± 0.44 ^b^	9.16 ± 1.27 ^c^	1.49 ± 0.44 ^a^	11.97 ± 1.46 ^d^
**Total Fiber**		15.10 ± 1.18 ^b^	53.89 ± 3.34 ^d^	11.72 ± 0.74 ^a^	24.18 ± 1.62 ^c^

Values with different lowercase letters in the same row indicate significant differences among samples (*p* < 0.05).

**Table 2:** HPLC-DAD-ESI/MS analysis of free (FP) and bound (BP) phenolic fractions of wheat grain (WG), oat grain (OG), wheat bran (WB) and oat bran (OB). Results were expressed in (μg g^−1^ d.m.).

	WG	WB	OG	OB
**SOLUBLE FRACTION**				
Ferulic acid	25.40 ± 1.10 ^a^	51.93 ± 2.69 ^b^	N.D.	N.D.
Caffeic Acid-4-Glucoside	17.69 ± 3.80 ^a^	67.33 ± 1.71 ^d^	22.39 ± 0.11 ^b^	30.45 ± 1.19 ^c^
Isovitexin 8-*C*-β-glucoside (Vicenin-2)	0.00 ± 0.00 ^a^	36.92 ± 0.68 ^d^	6.99 ± 0.23 ^b^	7.81 ± 0.10 ^c^
Apigenin-6/8-*C*-pentoside-8/6-*C*-hexoside I	19.96 ± 7.00 ^a^	15.56 ± 0.85 ^a^	11.54 ± 8.36 ^a^	15.55 ± 6.53 ^a^
Apigenin-6/8-*C*-pentoside-8/6-*C*-hexoside II	12.66 ± 6.06 ^c^	7.24 ± 0.00 ^b^	0.00 ± 0.00 ^a^	0.00 ± 0.00 ^a^
Apigenin-6/8-*C*-pentoside-8/6-*C*-hexoside III	12.09 ± 1.09 ^c^	0.00 ± 0.00 ^a^	5.29 ± 0.74 ^b^	4.26 ± 1.51 ^b^
Apigenin-8-*C*-glucoside (Vitexin)	115.97 ± 0.06 ^c^	551.05 ± 28.50 ^d^	24.74 ± 1.90 ^a^	49.63 ± 1.59 ^b^
1-Acetoxy pinoresinol	0.00 ± 0.00 ^a^	40.42 ± 1.88 ^b^	0.00 ± 0.00 ^a^	0.00 ± 0.00 ^a^
Daidzein	0.00 ± 0.00 ^a^	27.39 ± 1.54 ^a^	29.17 ± 0.11 ^a^	28.72 ± 1.33 ^a^
Apigenin-6-*C*-arabinoside-8-*C*-hexoside I	24.95 ± 2.62 ^b^	0.00 ± 0.00 ^a^	0.00 ± 0.00 ^a^	0.00 ± 0.00 ^a^
Apigenin-6-*C*-arabinoside-8-*C*-hexoside II	39.52 ± 0.82 ^b^	0.00 ± 0.00 ^a^	0.00 ± 0.00 ^a^	0.00 ± 0.00 ^a^
Avenanthramide K	0.00 ± 0.00 ^a^	0.00 ± 0.00 ^a^	6.15 ± 0.19 ^b^	8.16 ± 0.59 ^c^
Glycosilated/Acetylated-3′,4′,5′-trihydroxy-3,7-dimethylflavone	0.00 ± 0.00 ^a^	0.00 ± 0.00 ^a^	11.47 ± 0.62 ^b^	8.16 ± 0.26 ^a^
Apigenin-6-*C*-galactosyl-8-*C*-glucosyl-*O*-glucuropyranoside	15.63 ± 0.65 ^b^	56.11 ± 5.29 ^c^	0.00 ± 0.00 ^a^	0.00 ± 0.00 ^a^
Avenanthramide 2c	0.00 ± 0.00 ^a^	0.00 ± 0.00 ^a^	17.28 ± 0.24 ^c^	13.67 ± 0.01 ^b^
Avenanthramide 2p	0.00 ± 0.00 ^a^	0.00 ± 0.00 ^a^	32.28 ± 0.32 ^c^	17.23 ± 0.86 ^b^
Avenanthramide 2f	0.00 ± 0.00 ^a^	0.00 ± 0.00 ^a^	27.29 ± 0.00 ^b^	24.59 ± 1.60 ^a^
**Total**	**283.88 ± 0.50 ^c^**	**853.94 ± 39.08 ^d^**	**194.60 ± 10.53 ^a^**	**208.23 ± 15.56 ^b^**
**INSOLUBLE FRACTION**				
Medioresinol	233.48 ± 0.69 ^c^	891.66 ± 54.60 ^d^	126.23 ± 4.22 ^b^	76.22 ± 7.18 ^a^
Ferulic acid	3776.53 ± 4.74 ^b^	16,936.18 ± 1055.39 ^c^	1165.71 ± 5.63 ^a^	1769.94 ± 181.73 ^b^
Dihydroferulic Acid I	47.35 ± 0.36 ^c^	0.00 ± 0.00 ^a^	24.64 ± 5.44 ^a^	36.86 ± 1.98 ^b^
Dihydroferulic Acid II	188.41 ± 1.48 ^b^	333.62 ± 36.69 ^c^	86.65 ± 42.89 ^a^	88.08 ± 3.63 ^a^
Dihydroferulic Acid III	444.07 ± 0.07 ^c^	1215.03 ± 162.10 ^d^	190.58 ± 5.15 ^b^	182.94 ± 6.36 ^a^
**Total**	**4689.84 ± 19.33 ^c^**	**19,376.57 ± 911.20 ^d^**	**1593.81 ± 52.07 ^a^**	**2154.04 ± 200.88 ^b^**

Values with different lowercase letters in the same row indicate significant differences among samples (*p* < 0.05).

## Data Availability

Not applicable.

## Supplementary Materials

The following are available online at https://www.mdpi.com/2304-8158/10/1/115/s1, Supplementary Table S1. Correlation analysis of antioxidant parameters (DPPH, ORAC, ABTS, FRAP, and RACI). Supplementary Figure S1. HPLC phenolic profile of soluble and insoluble compounds of wheat and oat samples. (A) Wholegrain wheat soluble extract at 290 nm; (B) Wholegrain oat soluble extract at 290 nm. (C) Wholegrain wheat insoluble extract 320 nm. (a) whole The proposed phenolic compounds were numbered by the elution order of the chromatographic peak: 1. Caffeic acid-4-glucoside, 2. Isovitexin 8-C-β-glucoside (Vicenin-2), 3. Apigenin-6/8-C-pentoside-8/6-C-hexoside I, 4. Apigenin-6/8-C-pentoside-8/6-C-hexoside II, 5. Apigenin-6/8-C-pentoside-8/6-C-hexoside III, 6. Apigenin-8-C-glucoside (Vitexin), 7. 1-Acetoxy pinoresinol 8. Daidzein, 9. Apigenin-6-C-arabinoside-8-C-hexoside I; 10. Apigenin-6-C-arabinoside-8-C-hexoside II, 11. Avenanthramide K, 12. Glycosylated/Acetylated-3′,4′,5′-trihydroxy-3,7-dimethylflavone, 13. Ferulic acid 14. Apigenin-6-C-galactosyl-8-C-glucosyl-O-glucuropyranoside 15. Avenanthramide 2c; 16. Avenanthramide 2p; 17. Avenanthramide 2f, 18. Medioresinol, 19. Dihydroferulic Acid I, 20. Dihydroferulic Acid II, 21. Dihydroferulic Acid III. Supplementary Figure S2. α-Tocopherol content of wheat grain (WG), oat grain (OG) and their brans weight bran (WB) and oat bran (OB). Results were expressed µg 100 g^−1^ of lipid. Columns with different small letters show significant differences between samples (*p* < 0.05).
