# Supplementary material for: Wheat and Oat Brans as Sources of Polyphenol Compounds for Development of Antioxidant Nutraceutical Ingredients
Source: Foods. 2021 Jan 7;10(1):115. doi: 10.3390/foods10010115 (PMC7828044; doi:10.3390/foods10010115)
Supplement: Supplementary file 1 [file foods-10-00115-s001.zip › foods-1029989-supplementary.pptx]

## Slide 1
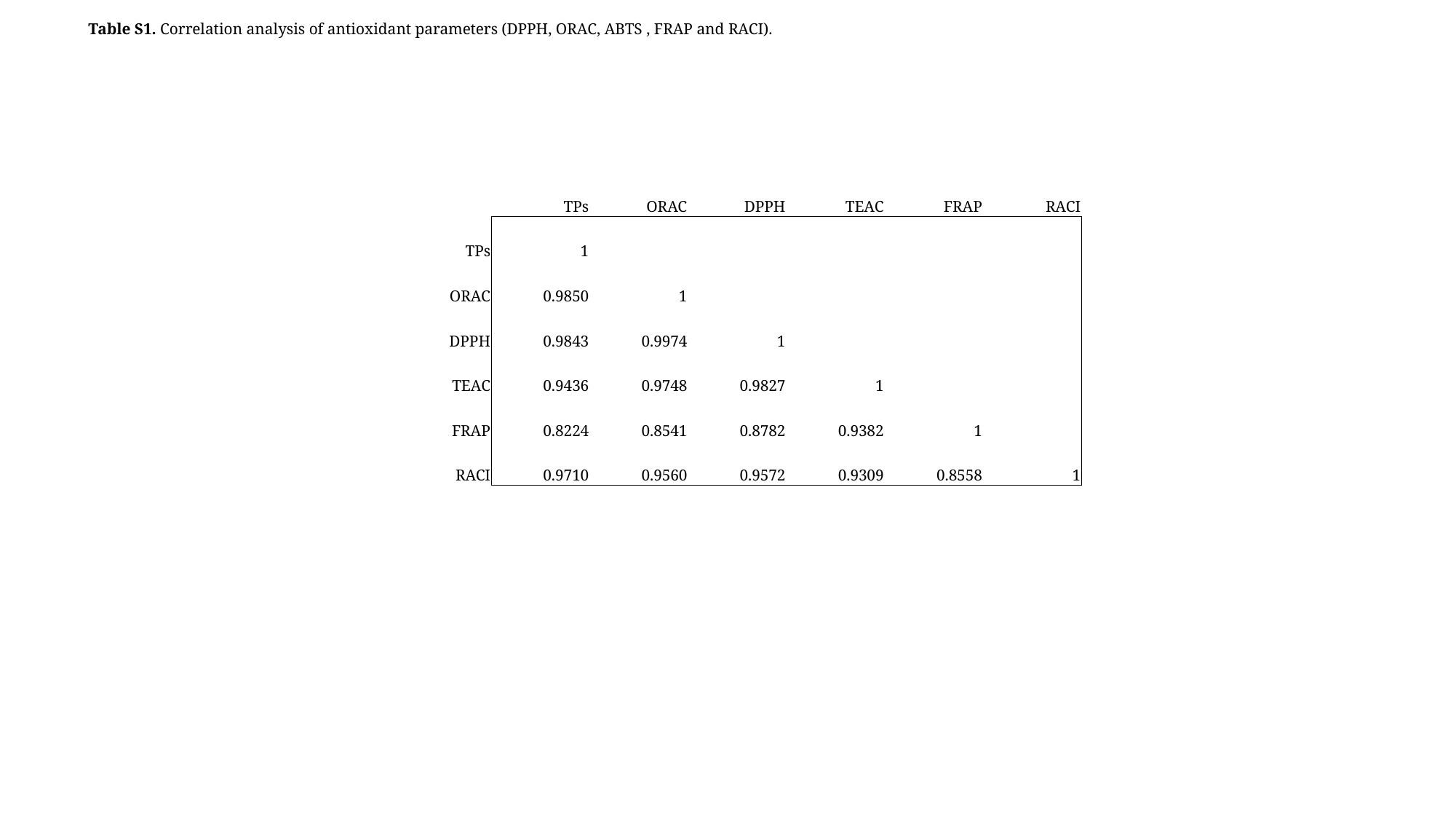

Table S1. Correlation analysis of antioxidant parameters (DPPH, ORAC, ABTS , FRAP and RACI).
| | TPs | ORAC | DPPH | TEAC | FRAP | RACI |
| --- | --- | --- | --- | --- | --- | --- |
| TPs | 1 | | | | | |
| ORAC | 0.9850 | 1 | | | | |
| DPPH | 0.9843 | 0.9974 | 1 | | | |
| TEAC | 0.9436 | 0.9748 | 0.9827 | 1 | | |
| FRAP | 0.8224 | 0.8541 | 0.8782 | 0.9382 | 1 | |
| RACI | 0.9710 | 0.9560 | 0.9572 | 0.9309 | 0.8558 | 1 |

## Slide 2
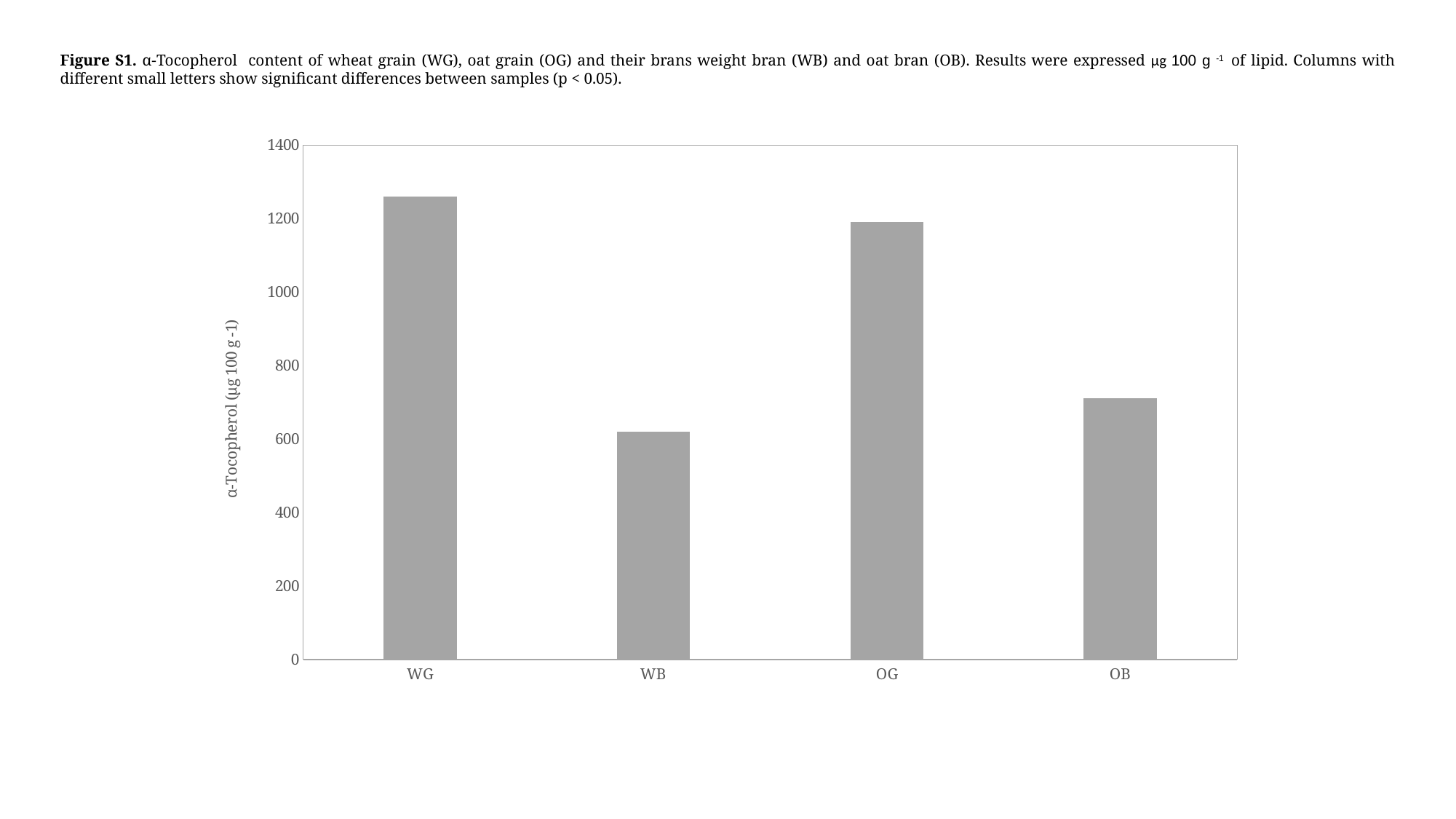

Figure S1. α-Tocopherol content of wheat grain (WG), oat grain (OG) and their brans weight bran (WB) and oat bran (OB). Results were expressed µg 100 g -1 of lipid. Columns with different small letters show significant differences between samples (p < 0.05).
### Chart
| Category | |
|---|---|
| WG | 1260.1294528143958 |
| WB | 619.7916084664588 |
| OG | 1190.4570687283667 |
| OB | 710.7179197186367 |

## Slide 3
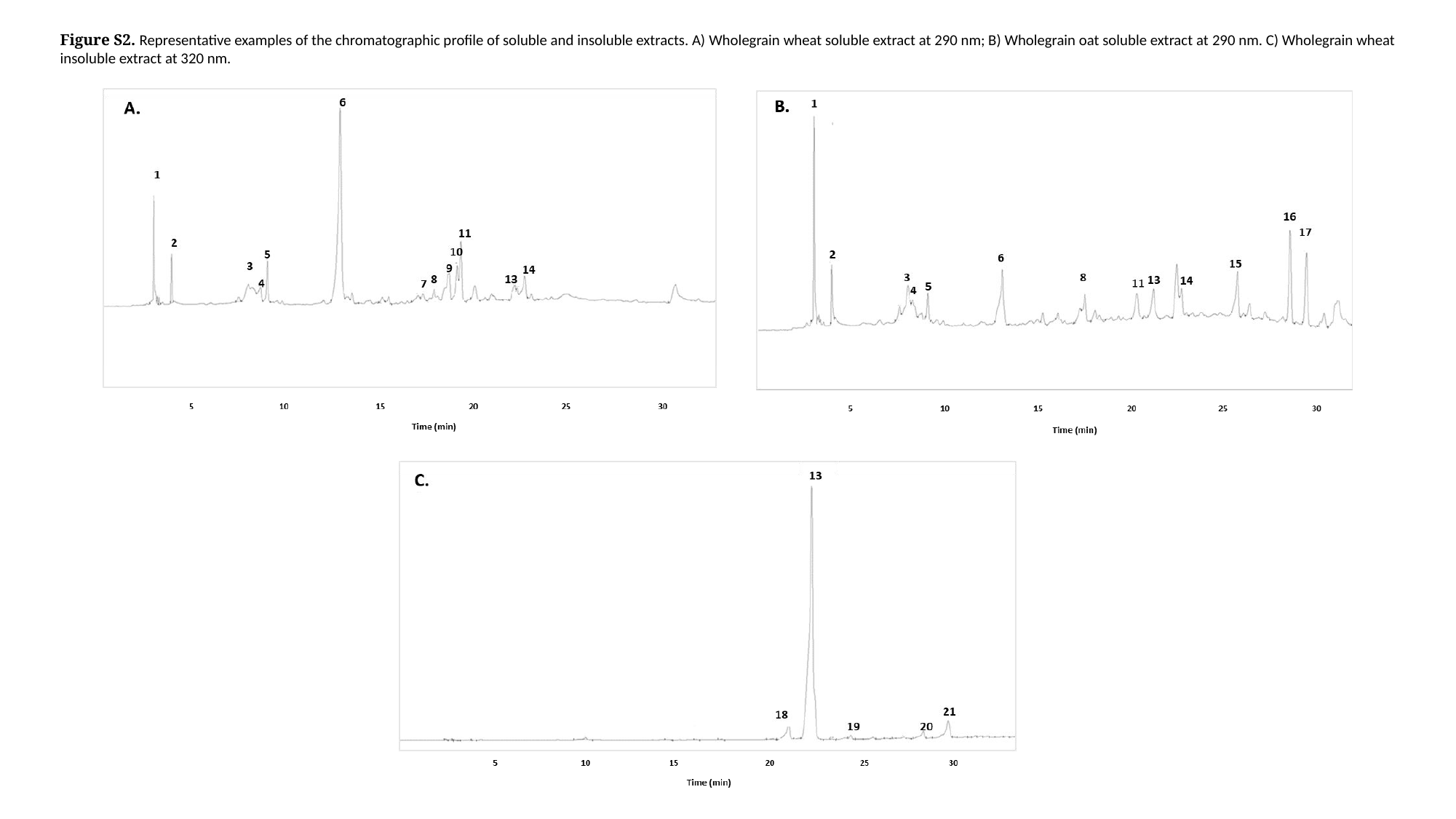

Figure S2. Representative examples of the chromatographic profile of soluble and insoluble extracts. A) Wholegrain wheat soluble extract at 290 nm; B) Wholegrain oat soluble extract at 290 nm. C) Wholegrain wheat insoluble extract at 320 nm.
